# Psychologic Study of Criminals

**Published:** 1916-04

**Authors:** 


**Psychologic Study of Criminals.** C. S. Rossy, using the Yerkes-Bridges point scale, Mass. State Board of Insanity Bull.

No. 17, reports as follows on 300 examinations in the State Prison: 22% feeble-minded, 9.6% border line cases, 3.3% presumably psychotic. The 22% are custodial cases.

